# Genomic epidemiology of Iranian *Bordetella pertussis*: 50 years after the implementation of whole cell vaccine

**DOI:** 10.1080/22221751.2019.1665479

**Published:** 2019-09-22

**Authors:** Azadeh Safarchi, Sophie Octavia, Vajihe Sadat Nikbin, Masoumeh Nakhost Lotfi, Seyed Mohsen Zahraei, Chin Yen Tay, Binit Lamichhane, Fereshteh Shahcheraghi, Ruiting Lan

**Affiliations:** a Pertussis Reference Laboratory, Department of Bacteriology, Pasteur Institute of Iran, Tehran, Islamic Republic of Iran; b School of Biotechnology and Biomolecular Sciences, University of New South Wales, Sydney, Australia; c Centre for Communicable Disease Control, Ministry of Health and Medical Education, Tehran, Islamic Republic of Iran; d Pathology and Laboratory Medicine, University of Western Australia, Perth, Australia

**Keywords:** *Bordetella pertussis*, Iran, whole genome sequencing, genomics, adaptation and evolution

## Abstract

Pertussis caused by *Bordetella pertussis,* remains a public health problem worldwide, despite high vaccine coverage in infants and children in many countries. Iran has been using whole cell vaccine for the last 50 years with more than 95% vaccination rate since 1988 and has experienced pertussis resurgence in recent years. Here, we sequenced 55 *B. pertussis* isolates mostly collected from three provinces with the highest number of pertussis cases in Iran, including Tehran, Mazandaran, and Eastern-Azarbayjan from the period of 2008-2016. Most isolates carried *ptxP3*/*prn2* alleles (42/55, 76%), the same genotype as isolates circulating in acellular vaccine-administrating countries. The second most frequent genotype was *ptxP3*/*prn9* (8/55, 14%). Only three isolates (5%) were *ptxP1*. Phylogenetic analysis showed that Iranian *ptxP3* isolates can be divided into eight clades (Clades 1-8) with no temporal association. Most of the isolates from Tehran grouped together as one distinctive clade (Clade 8) with six unique single nucleotide polymorphisms (SNPs). In addition, the *prn9* isolates were grouped together as Clade 5 with 12 clade-supporting SNPs. No pertactin deficient isolates were found among the 55 Iranian isolates. Our findings suggest that there is an ongoing adaptation and evolution of *B. pertussis* regardless of the types of vaccine used.

## Introduction

Despite high vaccine coverage in many countries, pertussis, also known as whooping cough is still reported in all age groups with the highest severity in infants and young children [1–3]. Pathogen adaptation through genetic changes has been shown to be one of the key roles in the re-emergence of pertussis, especially in countries were acellular vaccines (ACV) were used which commonly contained three (pertussis toxin (Ptx), pertactin (Prn), and filamentous haemagglutinin (Fha)) or five components (additional fimbriae Fim2 and Fim3) [1,3,4–6]. It has been shown that currently predominant *B. pertussis* isolates in many countries harbour non-ACV-antigen encoding alleles. The most common allelic profile worldwide, *ptxA1/ptxP1/prn1,* has changed to *ptxA1/ptxP3/prn2* [7]. *ptxP3*, the pertussis toxin (Ptx) promoter allele has been shown to be associated with higher virulence [5] and *ptxP3* isolates have a better fitness in animal studies [8].

While whole cell vaccine (WCV) has been replaced by ACV in many countries [2], it is still used in Iran since the 1950s for all scheduled doses in infants at 2, 4 and 6 months of age as well as two boosters at 18 months of age and six years old children [9,10]. Since 1988 vaccine coverage has increased to more than 95%, pertussis cases dramatically has declined from 40 cases per 100,000 in 1979 to 1.12 per 100,000 of population in 2011 [11,12]. However, increased pertussis notification rate has been reported in Iran since 2007 peaking in 2013 with 1415 suspected cases per 100,000 during the 2012–2014 pertussis epidemic [13–15]. The introduction of PCR based diagnosis, incomplete vaccination and the new guidelines and policies implemented in 2009 by Iran Centre for Communicable Disease Control that all suspected and confirmed cases must be notified and samples must be sent to the reference laboratory may have contributed to the increased number of reported pertussis cases in Iran [16].

In this study we analysed a selected set of *B. pertussis* isolates from 2008 to 2016 from nine provinces in Iran, especially from provinces with high number of pertussis cases to determine the evolutionary characteristics of Iranian *B. pertussis* using whole genome sequencing (WGS). Our aim was to expand our knowledge of the evolutionary forces, the genetic loci undergoing selective pressure and how *B. pertussis* may be evolving in response to WCV vaccine pressure in a country where WCV has been used in the last 50 years.

## Methods and materials

### Bacterial isolates and whole genome sequencing

**Figure 1.**
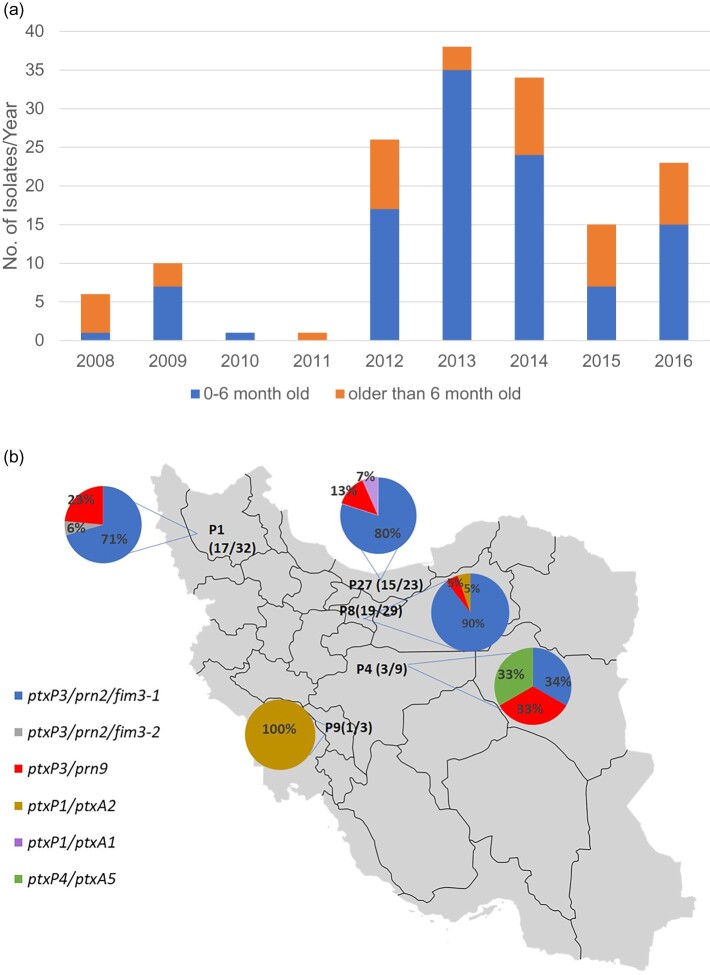
Basic statistics of isolates studied. (A) Distribution of isolates by year and vaccination status. A total of 154 *B. pertussis* isolates were available from the Reference laboratory. The graph shows their distribution by year and the proportion by the age of patients during 2008–2016. (B) The distribution of selected isolates and their ACV antigen gene profile results from different provinces. P1: Eastern-Azarbayjan, P4: Esfahan, P8: Tehran, P9: Charmahal, P27: Mazandaran. Numbers in the parentheses represent the number of selected isolates out of the total number of isolates available for each province.

A total of 154 clinical isolates from 2008 to 2016 from different provinces of Iran were available in Pasteur Institute of Iran as a reference diagnostic laboratory (Figure 1 (A)). Most of these isolates (107/154, 69.48%) were from patients aged up to 6-month-old and 40.26% (62/154), 32.46% (50/154) and 27.27% (42/154) were unvaccinated, partially or fully vaccinated respectively. Isolates were collected from 24 out of 31 provinces of which three provinces had more than 20 isolates including Eastern-Azarbayjan, Tehran and Mazandaran with 32, 29 and 23 isolates respectively.

We selected a total of 55 currently circulating Iranian *B. pertussis* isolates from 2008 to 2016 based on year and province of isolation. Amongst these 55 *B. pertussis* isolates, 51 were selected from three provinces which had the highest number of collected samples, including Tehran (Province-8), Mazandaran (northern province, Province-27) and Eastern-Azarbayjan (north-eastern province, Province-1) with 19, 15 and 17 isolates, respectively (Figure 1(B)). Thirty-two of the 51 isolates were from the 2012–2014 epidemic (Supplementary File-1). Additionally, four isolates from two provinces located in the centre of Iran (IR141, IR142, IR164 from Esfahan (Province-4), and IR138 from Charmahal (Province-9)) were added to have a better picture of geographical relationship of the isolates (Figure 1(B)).

DNA was extracted from bacterial culture as described previously [17]. The genomes were sequenced using 150 bp paired-end Illumina NextSeq sequencing with a minimum coverage of 150-fold. The raw reads were submitted to GeneBank under BioProject No. PRJNA450302.

### Data analysis

The distribution of Insertion sequence (IS) elements and SNP calling was performed and variation in genes encoding ACV antigens (*prn*, *ptxA, ptxP, fim2* and *fim3)* was investigated as described previously [17]. We detected SNPs and short insertion and deletions (indels) with less than 100 bp as previously described using Burrows–Wheeler Alignment (BWA) tools (v0.7.12), SAMtools (v0.1.19) and progressiveMauve (v2.3.1) [17–20]. Prokka (v1.12) and Roary (v3.11.2) were used for genome annotation and defining the core genomes, respectively [21,22]. *de novo* assembly was performed for all sequencing data to assemble reads into contigs using SPAdes (v3.7.0) [23]. Contigs were also aligned to *B. pertussis* strains Tohama I (GenBank Accession No. BX470248) and CS (Accession No. CP002695) as references to generate comparable data using progressiveMauve [18].

Phylogenetic analysis was conducted using MEGA (v7) [24]. The maximum parsimony algorithm was used to construct phylogenetic tree. Tree-Bisection-Reconnection (TBR) was used to search optimal trees. Bootstrap analysis was based on 500 replicates and *B. pertussis* Tohama I used as outgroup.

## Results

### ACV vaccine antigen gene allele profiles

Typing of ACV antigen genes, *ptxP*, *ptxA*, *prn*, *fim2* and *fim3* among the 55 selected isolates from 2008 to 2016 revealed that *ptxP3/ptxA1/fim2-1* isolates were predominant (51/55, 92.7%). Out of the 51 *ptxP3* isolates, 50 isolates carried *fim3-2* (also denoted as *fim3B*) allele and only one had *fim3-*1 (also denoted as *fim3A*) [25,26]. Isolates with the allele profile *ptxP3/ptxA1/prn2/fim3- 1*were predominant among *ptxP3* isolates (42/51, 82.3%). We found a new profile of *ptxP3/ptxA1/prn9/fim3-2* with eight isolates (8/51,15.68%) as the second most frequent profile. All *prn9* isolates were isolated after 2013 and rose from 12.5% (2/15) in 2013 to 27% (3/11) in 2016. These isolates were not reported previously in Iran and collected from different provinces showing the expansion of *prn9* isolates within the country (Figure 1(B)). There were only four *non-ptxP3* isolates, one, IR142, harboured a *ptxP4* allele (full profile: *ptxP4*/*ptxA5*/*prn6*/ *fhaB2*/ *fim2-1*/ *fim3-1*), and three were *ptxP1* which were further differentiated by *ptxA* allele into *ptxP1/ptxA1*/*fim2-1*/*fim3-1/ prn1* (one isolates, IR146) and *ptxP1/ptxA2*/*fim2-1*/ *fim3-1/ prn1* (two isolates, IR136 and IR138).

### Phylogeny of Iranian B. pertussis isolates

**Figure 2.**
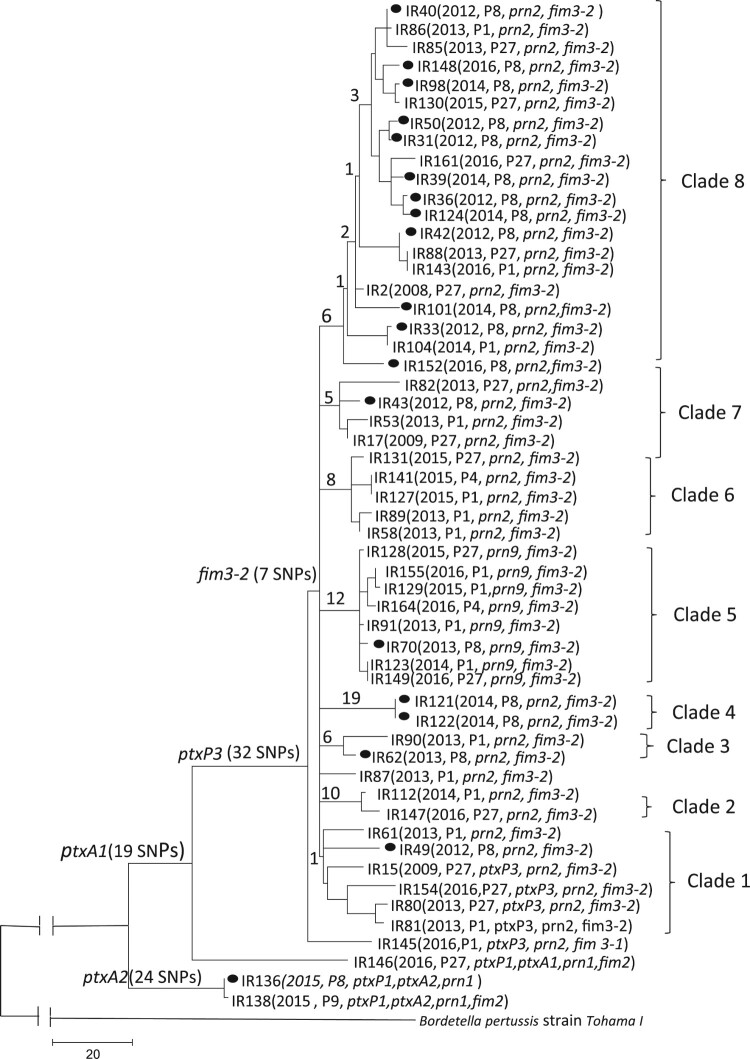
Maximum parsimony tree of the 54 Iranian *B. pertussis* isolates. The tree was constructed based on 653 SNPs. The number on the internal and terminal branches corresponds to the number of SNPs supporting each branch. Iranian *ptxP3/ fim3-2* isolates were grouped into 8 clades as marked. The majority of *ptxP3* isolates collected from Tehran grouped together (12 out of 18) in clade 8. New profile of *ptxP3/ ptxA1/prn9* was defined as clade 5. Black circle on the tree represents the distribution of isolates collected from Tehran.

Phylogenetic analysis based on SNPs across the whole-genome differentiated the 55 isolates into three major lineages, 51 *ptxP3* isolates into one lineage, three *ptxP1* isolates into the second (Figure 2) and the outlier (IR142 with *ptxP4*/ *ptxA5*/ *prn6*/ *fhaB2* allele profile) which were omitted from the tree due to high number of SNPs and will be discussed later. There were 32 SNPs shared by all *ptxP3* Iranian isolates (Table 1) but none was unique to Iranian isolates, some of which were also reported among global non*-ptxP3* isolates [7]. All except one *ptxP3* isolate were grouped together as one lineage and all carried *fim3-2* allele with 7 SNPs supporting the node and were further divided into eight clades as Clade 1 to Clade 8 supported by 1, 10, 6, 19, 12, 8, 5 and 6 SNPs, respectively (Figure 2 and Table 1). There was no correlation between isolates in terms of year of isolation except for Clade 3 and Clade 4. There were only 2 isolates each in Clade 3 and Clade 4 which were obtained in 2013 and 2014, respectively. The 2012–2014 epidemic isolates were interspersed within and between clades with no temporal correlation. Interestingly, all eight *prn9* isolates were clustered as Clade 5 supported by 12 unique SNPs. Clade 8, supported by 6 SNPs, was the largest clade with 20 isolates, of which 12 (70%) were from Tehran. One isolate (IR87) collected in 2013 from Eastern-Azarbayjan was not grouped within any clades and defined as an ungrouped isolate. The two *ptxP1/ptxA2* isolates were collected in 2015 and were well separated from the *ptxP1/ptxA1* isolate (Figure 2).

**Table 1.** Single nucleotide polymorphism (SNP) and short insertion and deletions (indels) supporting the nodes and clades in phylogenetic analysis.

Branch/Clade	Provinces^a^	SNPs^b^	Nature of SNP and position in Tohama I^c^	Indels^b^
8	P1 (3), P8 (12), P27 (5)	Genic (5)	4 nsSNPs: BP0769, BP1245, BP2428, *glpK*	ND
			1 sSNPs: BP0612	
		Intergenic (1)	Intergenic area	
				
7	P1 (1), P8 (1), P27 (2)	Genic (5)	1 nsSNPs: BP3060	Deletion: BP1766p (1)
			4 sSNPs: BP2233, BP2428, BP2539, BP3329	
				
6	P1 (3), P4 (1), P27 (1)	Genic (8)	7 nsSNPs: BP1193, BP1362, BP1374(*fla FIII*), BP1924, BP2162, BP3843, BP3856	Deletion: BP0041p (1)
			1 sSNPs: BP0425	
				
5	P1 (4), P4 (1), P8 (1), P27 (2)	Genic (6)	6 sSNPs: BP0170, BP1322, BP1704, BP2162, BP2548, BP 3694	Deletion: intergenic area (12)
		Intergenic (6)	BP0070p, BP0170p, BP0359p, *bvgA/fhaB*p, BP3049p, BP3439 (*dnt*)*p*,	
				
4	P8 (2)	Genic (15)	10 nsSNPs: BP0043, BP0999 (*tkt*), BP1507, BP2293, BP2340, BP2882, BP3462, BP3518, BP3671, BP3680 (*glc*),	Insertion: BP0513 (12), BP0565p (1)
			5 sSNPs: BP1322, BP1858 (*aldH*), BP3459, BP3874 (*ptxB*) BP3484	
		Intergenic (4)	BP0018p, BP1054 (*prn*)*p*, BP2956p, one in intergenic area	
				
3	P1 (1), P8 (1)	Genic (5)	4 nsSNPs: BP0767 (*gltK*), BP1208, BP2987 (*dltA*), BP3570	ND
			1 sSNPs: BP3158 (*mmsA*)	
		Intergenic (1)	BP2224 (*bapB*)*p*	
				
2	P1 (1), P27 (1)		7 nsSNPs: BP0170 (rhlE), BP1240, BP1376 (*flaFV*), BP1732 (*ppiD*), BP1808 (*paaG*), BP2691, BP2780	ND
			3 sSNPs: BP0668, BP2624 (*guaA*), BP3474	
				
1	P1 (2), P8 (1), P27 (1)	Genic (1)	1 nsSNPs: BP0376 (*mreD*)	ND
				
*fim3-2* isolates	Different provinces	Genic (4)	4 nsSNPs: BP0646, BP1086, BP1568 (*fim3*), BP2366	
		Intergenic (3)	BP1157p, BP1711p, BP3806/BP3807p	
				
*ptxP3* isolates	Different provinces	Genic (25)	13 nsSNPs: BP0194, BP0292, BP0518, BP0854 (*nuoN*), BP0891, BP1090, BP1261, BP1471, BP2249 (*bscI*), BP2485	Deletions: BP0880 (8), BP2946 (31), *fim2*p (5)
			11 sSNPs: BP0184, BP0215 (*ppc*), BP0505, BP0507, BP0678 (*prfA*), BP1054 (*prn*), BP1227 (*radA*), BP1741, BP3630 (*rpsH*), BP3787 (*ptxC*)	
		Intergenic (7)	BP0032p/BP0033p, BP0049p, BP0499p/BP0500p, Intergenic, BP1035p, BP3062p, BP3783 (*ptxA*)*p*	
				
*ptxP1/ptxA2* isolates	P8 (1), P9 (1)	Genic (18)	10 nsSNPs: BP0082 (*bplL*), BP0724, BP1142, BP1296, BP1324 (*malA*), BP1881 (*fhaD*), BP2117, BP2467, BP3271, BP3385	Insertion: BP0192 (1), *cusC* (1), *copA* (5)
			8 sSNPs: BP0222 (*catJ*), BP0454, BP1037 (*cutE*), BP1142, BP1203, BP1952, BP2215(*ectA*), BP2235 (*bscC*)	Deletion: promoter region of *serA* (1) and BP2159 (1)
		Intergenic (6)	BPt15p, BP1036p, BP1364p, BP378p, BP378p, BP3698p, BP3698p	

^a^Province P1: Province number P1 (Eastern Azarbayjan), P4: Province number 4 (Esfahan), P8: Province number 8 (Tehran), P27: Province number 27 (Mazandaran). Number in brackets indicates number of isolates.

^b^The numbers in each column represents the number of isolates, SNPs or bases.

^c^nsSNP: non-synonymous SNPs, sSNP: Synonymous SNP, ND: not determined, *p*: promoter region

### SNPs or indels associated with virulence genes or potential adaptive evolution

We examined the SNPs and indels that may be associated with gene functions and play an adaptive role. From 654 SNPs found in this study (Supplementary File-2), a total of 310 SNPs were unique to one or more Iranian isolates which had not been reported previously. Nine nsSNP were from virulence associated genes including *sphB2*, *sphB3*, *tcfA*, *bscQ*, *vag8*, *fhaL*, *ompQ*, *brkA* and *ptxB* genes (Table 2). There are 11 nonsense (stop codon) SNPs, eight of which were unique to Iranian isolates (Table 2). A novel nonsense SNP in *fhaD* was found in IR136 and IR138, both of which were *ptxP1*/*ptxA2* isolates. *fhaD* encoding a chaperone protein is known to be an accessory gene which may affect the production and secretion of Fha and fimbriae in *B. pertussis* [27].

**Table 2.** Unique non-synonymous SNPs in virulence associated genes or nonsense SNPs that were only found in Iranian *B. pertussis* isolates.

Position in Tohama I	strains	Base mutation	AA (Ref.)	AA_SNP	Locus	Gene	Protein	Functional category	Up/Down regulated^a^
30440	IR124	C →T	W	STOP	BP0027	–	MaoC family protein	Miscellaneous	
1897065	Clade 2	G→A	Q	STOP	BP1808	*paaG*	probable enoyl-CoA hydratase	Small molecule degradation	
1980668	*ptxA2* strains	G→A	W	STOP	BP1881	*fhaD*	chaperone protein	Virulence-associated genes	
2355697	IR154	G→A	Q	STOP	BP2229	–	putative inner membrane transport	Transport/binding proteins	
2515775	IR40, IR85, IR86	C→A	Y	STOP	BP2376	–	Conserved hypothetical protein	Conserved hypothetical	
2545945	IR88, IR143	G→A	W	STOP	BP2405	–	putative cytochrome p450 oxidoreductase	Energy metabolism	+
3320437	IR33, IR104	C→T	W	STOP	BP3117	–	putative restriction endonuclease	Macromolecule synthesis/modification	
3403673	IR161	G→A	Q	STOP	BP3191	–	putative acyl-CoA dehyrdogenase	Miscellaneous	
513355	IR147	G→A	S	N	BP0500	*bteA*	hypothetical protein	Virulence-associated genes	+
1163496	IR88, IR143, IR42	G→A	A	T	BP1110	*sphB3*	serine protease	Virulence-associated genes	+
2368953	IR80, IR81	C→T	A	T	BP2241	*bscQ*	putative type III secretion protein	Virulence-associated genes	
2369042	IR87	A→G	V	A	BP2241	*bscQ*	putative type III secretion protein	Virulence-associated genes	
2380907	IR49	G→A	G	D	BP2258	–	hypothetical protein	Virulence-associated genes	
3089988	IR124	C→T	P	L	BP2907	*fhaL*	adhesin	Virulence-associated genes	
3092423	IR147	G→A	A	T	BP2907	*fhaL*	adhesin	Virulence-associated genes	
3613994	IR129, IR155, IR164	G→A	A	V	BP3405	*ompQ*	outer membrane porin protein OmpQ	Virulence-associated genes	+

aBased on study by King et al. [5].

Some clade dividing SNPs may be clade specific adaptive changes including changes in the promoter or coding region of virulence associated genes or metabolic genes. Three of the 12 SNPs supporting the grouping of *prn9* isolates as Clade 5 (Table 1) were of interest: one SNP located in the promoter region of the *dnt* (BP3439) gene encoding dermonecrotic toxin, one in the promoter region of *fhaB* and *bvgA* genes and the third a nsSNP located in BP2548 encoding a histidine kinase. The mutation in the *fhaB* promoter region was also found in the Fha/Prn negative *B. pertussis* strain isolated in 2009 in France [28]. However, it remains unknown if this mutation leads to non-FhaB/Prn expression since it was identified in one UK isolate (UK98) which appeared to be Fha and Prn positive [7,29–31]. The nsSNP in BP2548 which encodes a two-component system histidine kinase and has not been reported previously. One of the six SNPs supporting Clade 8 was a nsSNP located in the *glpK* gene which encodes a glycerol kinase; a key enzyme in the regulation of glycerol uptake and metabolism.

### Insertion sequence elements

A total of 19 IS elements due to the IS*481*F or IS*481*R (F and R denote insertion in the forward or reverse direction) that were absent in Tohama I were identified (Supplementary File-3), all of which have been reported previously [17,30]. One IS in BP1446 encoding enoyl-CoA hydratase was found in all Clade 2 isolates. Two were found in Clade 4 with an IS*481*F in BP2232 encoding hypothetical protein and an IS*481*R in BP3588 encoding acetylornithine deacetylase. Furthermore, two IS*481*R insertions located in BP1591 and BP3764 were found only in *ptxA2* isolates, IR136 and IR138. IR142 was compared with Tohama I separately and 22 IS disruptions were found for this isolate, of which, 19 were unique. It is of interest that IR142 shared with *B. pertussis* 18323 an IS*481*R in the *fhaL* gene encoding a putative protein homologous to Fha that led to a pseudogene [32]. No IS disruptions were identified in *prn* or other ACV-antigen genes.

### Region of differences in Iranian B. pertussis genomes

Current circulating *ptxP3* isolates appear to have lost clusters of genes, also known as regions of difference (RD) including RD3 (BP0910A-BP0937), RD5 (BP1134-BP1141) and RD10 (BP1947-1968) [33–35]. We found 59 loci with more than two genes deleted in one or more isolates (Supplementary File-4) including RD3 deleted in all 55 isolates while RD5 was deleted in all isolates except IR142. Only BP1134 and BP1142 of RD5 were deleted in IR142. RD10 was absent in all *ptxP3* isolates whereas only BP1947 and BP1968 of RD10 were deleted in all non-*ptxP3* isolates. A set of 20 genes (BP1157-BP1177), of which six encoding cell surface proteins, were deleted in all *prn9* isolates and IR146 (*ptxP1/ptxA2/prn1*). Two large fragments, containing 18 (BPTD2835-BPTD2852) and 21 (BPTD0387 to BPTD0407) genes, that were absent in Tohama I but present in the other reference Chinese CS strain and many other isolates [36,37], were also present in all Iranian isolates.

### The evolutionary relationships of Iranian B. pertussis from the global perspective

**Figure 3.**
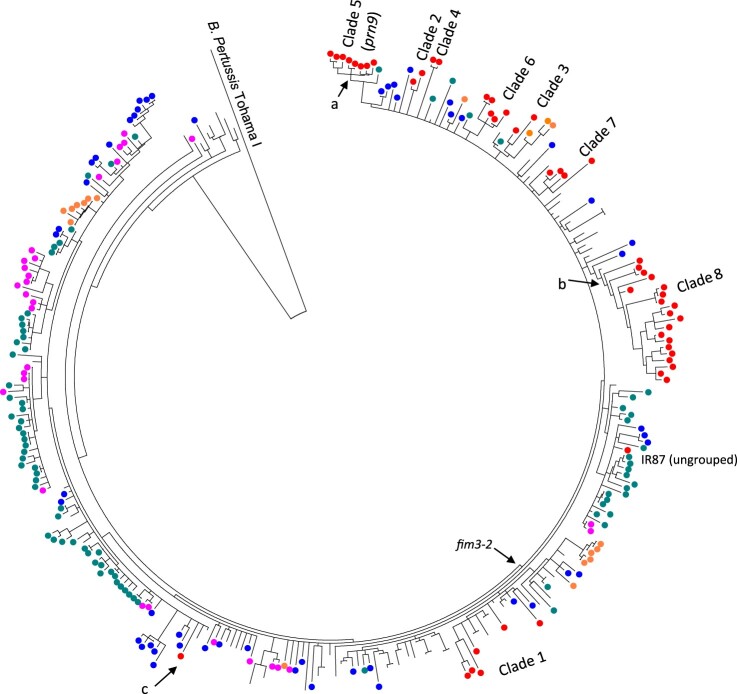
Phylogenetic relationships of Iranian *ptxP3 B. pertussis* isolates with global isolates. The tree was inferred using maximum parsimony method using *B. pertussis* Tohama I as an outgroup. A total of 304 *ptxP3* isolates from different continents were included (Red: Iran, Blue: The USA, Green: UK, Pink: Australia) (details of the isolates are available in Supplementary File-1). Iranian isolates showed the same clustering as clade 2–8 except isolates in clade 1 that were interspersed with isolates from other countries. Clade 5 had 12 clade supporting SNPs, seven of which were shared with UK98 *prn2* isolate (green circle) and two nsSNPs positioned in BP2548 and BP3694 and one mutation in the promoter of BP0359 were still unique to Iranian isolates (Node marked with **a**). For 6 clade 8 specific SNPs, five were shared with one Canadian isolate (B062, isolated in 2001) and one nsSNPs located in B1245 was still unique to Iranian isolates (Node marked with **b**). The only *ptxP3/fim3-1* Iranian isolate (IR145) was grouped with *fim3-1* isolates from USA (Node marked with c).

To place the Iranian isolates in a global context, we determined the genetic relationships of the 51 Iranian *B. pertussis ptxP3* isolates with 252 *ptxP3* genomes representing 18 countries from 1988 to 2016 from multiple studies [7,17,29,30] (Figure 3). *ptxP3* isolates were distributed among two large lineages demarcated by *fim3* alleles as reported previously [7,17,29]. The only *fim3-1* isolate, IR145, grouped with *fim3-1* isolates from the USA. Clade 1 isolates were distributed among global isolates isolated between 1997–2003 suggesting multiple parallel spread of *B. pertussis* isolates into Iran. Clade 2 to 8 were still clustered together with each clade being separately close to isolates mainly from the USA. *prn9* isolates as Clade 5 were grouped with one *prn2* isolate from the UK (UK98 isolated in 2012). From 12 unique SNPs for *prn9* isolates as clade 5, seven were shared with UK98 and two nsSNPs positioned in BP2548 and BP3694 and one mutation in promoter of BP0359 were still unique to Iranian isolates. Clade 8 as largest Iranian clade grouped with one Canadian isolate (B062, isolated in 2001) and from 6 unique SNPs for this clade 1 nsSNP located in B1245 was still unique to Iranian isolates. Finally, the ungrouped isolate, IR87, was grouped with UK *fim3-2* isolates.

**Figure 4.**
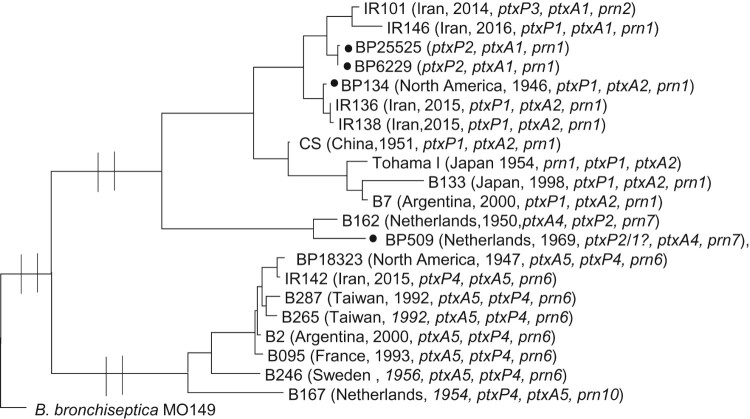
Maximum Parsimony Phylogeny using 2792 SNPs to analyse the relationship between old *B. pertussis* isolates, vaccine seed isolates and recent isolates from Iran. *B. bronchiseptica* MO149 used as an outgroup. Isolated marked with black circle are vaccine seeds used in WCV manufactured by Serum Institute of India that is used for immunization in Iran. BP509 and BP134 were also used in WCV in the years when Iran manufactured and used its own vaccine.

The relationship of IR142, which stands as a distinct lineage from the remaining 54 Iranian *B. pertussis* isolates was investigated. The phylogenetic analysis revealed that IR142 was more closely related to some old divergent isolates (seven isolates from different continents) from before 1950s reported by Bart *et al.* [7] and in particular to *B. pertussis* 18323 (Figure 4) with the same allelic profile. Furthermore, the relationship of four strains used as WCV vaccine seeds, BP134, BP509, BP6229 and BP25525, with recently collected isolates from Iran were also investigated [38]. The four strains have been used to manufacture the Indian DTwP, which has been used in Iran for immunization in recent years. BP509 and BP134 were also used as vaccine seeds from 1950 by Iran to manufacture its own DTwP [39]. Four recent Iranian isolates were selected for comparison including three *ptxP1* isolates (IR136, IR138 and IR146) which have almost the same allelic profile with three vaccine seed strains and one random *ptxP3* isolate (IR101) which was more distinct. The results revealed that BP509 with *ptxA4/prn7* genotype was more distinct than other vaccine seeds. The other three vaccine seed strains grouped together with the *ptxP1* isolates including IR136, IR138 and IR146 (Figure 4). As expected, all vaccine seed strains were distinct from the current circulating *ptxP3* isolate (IR101).

## Discussion

Iran has been using WCV for immunization against pertussis for more than 50 years and continues to use WCV with a high WCV coverage [10]. It encountered an increase in pertussis cases since 2007 with highest number in 2013 with 1415 suspected cases per 100,000 [15]. A previous PFGE study revealed that most isolates collected during 2009–2014 belonged to PFGE group 10 which is likely to be equivalent to the PFGE group SR11 in European countries [40]. Furthermore, ACV antigen gene typing of a small number of isolates showed that *ptxP3* isolates were predominant in Iran during 2008–2015 [40,41]. In this study we examined the genomic differences and relationships of *ptxP3* isolates collected in Iran with WCV immunization and compared with *ptxP3* isolates from countries with ACV vaccination. Our analysis of 55 Iranian *B. pertussis* isolates collected after the pertussis resurgence showed that Iranian *ptxP3* isolates were grouped into 8 clades, each had separately derived from or closely related to isolates from other countries including USA, UK and Canada, suggesting multiple importation of *B. pertussis* into Iran and then diversification and expansion of different clades in parallel.

The results further confirmed that the *ptxP3* strains have spread across the globe including WCV regions and can compete effectively against non-*ptxP3* strains in a WCV immunised population. This study is one of the few evolutionary genetic studies of *B. pertussis* in the middle east region where most of its neighbouring countries are also using WCV and our study underscores the importance of surveillance of *B. pertussis* in these countries.

The expansion of *ptxP3* strains has been suggested to be associated with the replacement of WCV by ACV although this hypothesis has been controversial [42]. In Poland where WCV has also been in use for decades, in 2000–2013, *ptxP1* isolates predominated with 77.5% [43]. However, in a more recent study, 61.5% of the isolates during 2010–2016 were found to be *ptxP3* and were attributed to the increasing use of ACV since 2013 for primary immunization [43–45]. The near complete replacement of the *B. pertussis* population in Iran by *ptxP3* strains where only WCV has been used further suggests that the expansion of *ptxP3* strains was not driven by ACV selection alone. In China where ACV has replaced WCV in 2012, both *ptxP3* and *ptxP1* strains circulate with predominance of *ptxP1*. However, the *ptxP1* strains have been found to be resistant to macrolides which are used for pertussis treatment and prophylaxis [46]. Macrolide resistance may have acted as selection pressure to maintain the *ptxP1 B. pertussis* population in China [46].

Comparative studies of *ptxP3* and *ptxP1* strains suggest that *ptxP3* strains can outcompete *ptxP1* strains in mice regardless whether the mice were immunised with ACV [8] and proteomics studies suggest that *ptxP3* strains may have become metabolically fitter [47]. Several studies have shown that some genes involved in amino acid biosynthesis regulation and energy metabolism are differentially expressed in *ptxP3* strains compared with *ptxP1* strains [5,47,48]. In this study 17 nsSNPs have been found in metabolic genes of which 14 were only in Iranian isolates. Three metabolic genes (BP1072 (*ppk*), BP2212 (*fhp*) and BP2405) have been reported to be upregulated in *ptxP3* strains [5], which suggests further potential adaptation. However, it is difficult to ascertain their effect as the mutations may be neutral or even mildly deleterious.

Continued adaptation by *ptxP3* strains is evident in virulence associated genes [5]. From 15 nsSNPs in 12 virulence associated genes found only in *ptxP3* strains, eight were only in Iranian isolates (Table 2), of which three were in genes (*bteA*, *sphB3*, *ompQ*) which have been reported to be upregulated in *ptxP3* strains [5]. There were two novel SNPs in the *bteA* gene and in its promoter. *bteA* (BP0500) is known to be a BvgAS-regulated gene and thought to play a crucial role in T3SS-mediated cell death [49]. It is also shown to be less sensitive to be regulated in *ptxP3* isolates in the presence of sulphate [50]. From six SNPs in *ptxP3* isolates that were in genes encoding ACV antigens (*fim3, fhaB, prn* and *ptxA, B* and *C*), three were nsSNPs and all have been reported previously.

A prominent feature of *B. pertussis* evolution in ACV immunised host populations is the emergence and expansion of pertactin deficient strains [4]. Prn deficient strains were first reported in France, and now in many countries including UK, Canada, US and Australia [25,26,51–53]. Australia and US reported the highest percentages of Prn deficient isolates with 89.7% of the isolates from 2013 to 2017 in Australia and >85% in 2013 in the US [26,54]. Multiple mechanisms of Prn non-expression were found with the majority of the Prn deficiency due to damage of the *prn* gene by IS*481* insertion at a hotspot in the *prn* structural gene [54]. All known mechanisms of *prn* inactivation were examined from the genome sequence data of the Iranian isolates and none of the Iranian isolates was found to be potentially pertactin negative. Since WCV contained far more antigens than the ACVs that contain pertactin, there may be less selection pressure and less advantage for pertactin deficient *B. pertussis* mutants to emerge in a WCV immunized population.

The Iranian *ptxP3* isolates were divided into multiple clades. Clades 5 and 8 are of particular interest. Clade 5 carried the *prn9* allele exclusively. *prn9* isolates were previously observed in Japan from 2008 to 2012 with low frequency [55] and sporadic isolations in other countries [56,57]. However, it is unknown whether Japanese *prn9* isolates were related to Iranian isolates or derived independently. It is possible that the increasing prevalence of *prn9* isolates in the WCV population were due to the pressure of WCV. *prn9* allele produces near the same amino acid sequence as *prn2* with one additional GGFGP repeat in region 1 of the *prn* gene. A global comparison also showed that all *prn9* isolates shared seven SNPs with a *prn2* UK isolate UK98, suggesting that *prn9* isolates may have arisen from a *prn2* background. The additional repeat might affect epitope structure as difference in epitopes between Prn2 and Prn1 alleles were found [58]. Clade 8 contained the highest number of isolates (20), mostly collected from Tehran (12/20). Clade 8 has 22 SNPs in one or more isolates that are located in genes or promoters reported to be upregulated in *ptxP3* isolates [5].

IR142 was closely related to *B. pertussis* strain 18322 that had diverged before the expansion of *B. pertussis* Tohama I lineage [7]. This divergent lineage has rarely been isolated in recent years [32, 34, 35]. Nevertheless, it is interesting to note that IR142 was isolated in 2015, suggesting that the lineage might still be circulating in Iran at a very low frequency [34,35].

Resurgence of pertussis in Iran may be associated with divergence between vaccine seeds and circulating strains. Iran used to administer the DTwP vaccine locally manufactured by Razi Serum Institute for decades [59]. Due to reports of low immunogenicity and high adverse reactions of the vaccine [39,60], the local vaccine was replaced by other commercial vaccines in 2009, mostly the Indian DTwP. Furthermore, the increase in the pertussis incidence in Iran in recent years may indicate low efficiency of the vaccines used to control the disease [11,12]. It was suggested that bacterial strains or the vaccine formulation in some batches of vaccines might be the reason behind these issues [59]. Comparison of the vaccine strains used in manufacturing the Iranian WCV (BP509 and BP134) and Indian WCV (BP509, BP134, BP25525 and BP6229) with currently circulating clinical isolates showed that none was close to the current predominant *ptxP3* isolates in Iran (Figure 4). BP509 that was used in both Iranian and Indian vaccines was more distinct compared to other vaccine seeds due to its allelic profile (*ptxA4/prn7*). BP134, also used in both Iranian and Indian vaccines was grouped with the now less predominant *ptxP1/ptxA2* isolates in Iran. These data may explain why the local DTwP vaccine regardless of the quality variation between vaccine batches was less effective to control pertussis in Iran and substitution of vaccine seeds with a currently predominant strain for vaccine production may provide a more effective vaccine. However, more studies are needed to ascertain the contribution of the divergence between vaccine seeds and currently circulating strains to pertussis resurgence in Iran.

In summary, this study found that the Iranian *B. pertussis* isolates from a WCV immunised population carried mostly the *ptxP3* allele similar to *B. pertussis* circulating in many countries where ACVs are used. The isolates were grouped into 8 clades which were independent importations from other countries and then local expansion. The Iranian ptxP3 *B. pertussis* population was not due to the emergence of a novel, hypervirulent clone or the expansion of a single lineage. These findings have important implications on the control of pertussis as current vaccines are not an effective measure to control the newly evolved *ptxP3* strains. Further epidemiological surveillance is important to monitor the evolutionary changes of the *B. pertussis* population to help develop more effective strategies to control pertussis.

## Supplementary Material

Supplemental Material
