# Supplementary figures and images for: Genomic epidemiology of Iranian Bordetella pertussis: 50 years after the implementation of whole cell vaccine
Source: Emerg Microbes Infect. 2019 Sep 22;8(1):1416–27. doi: 10.1080/22221751.2019.1665479 (PMC6764348; doi:10.1080/22221751.2019.1665479)

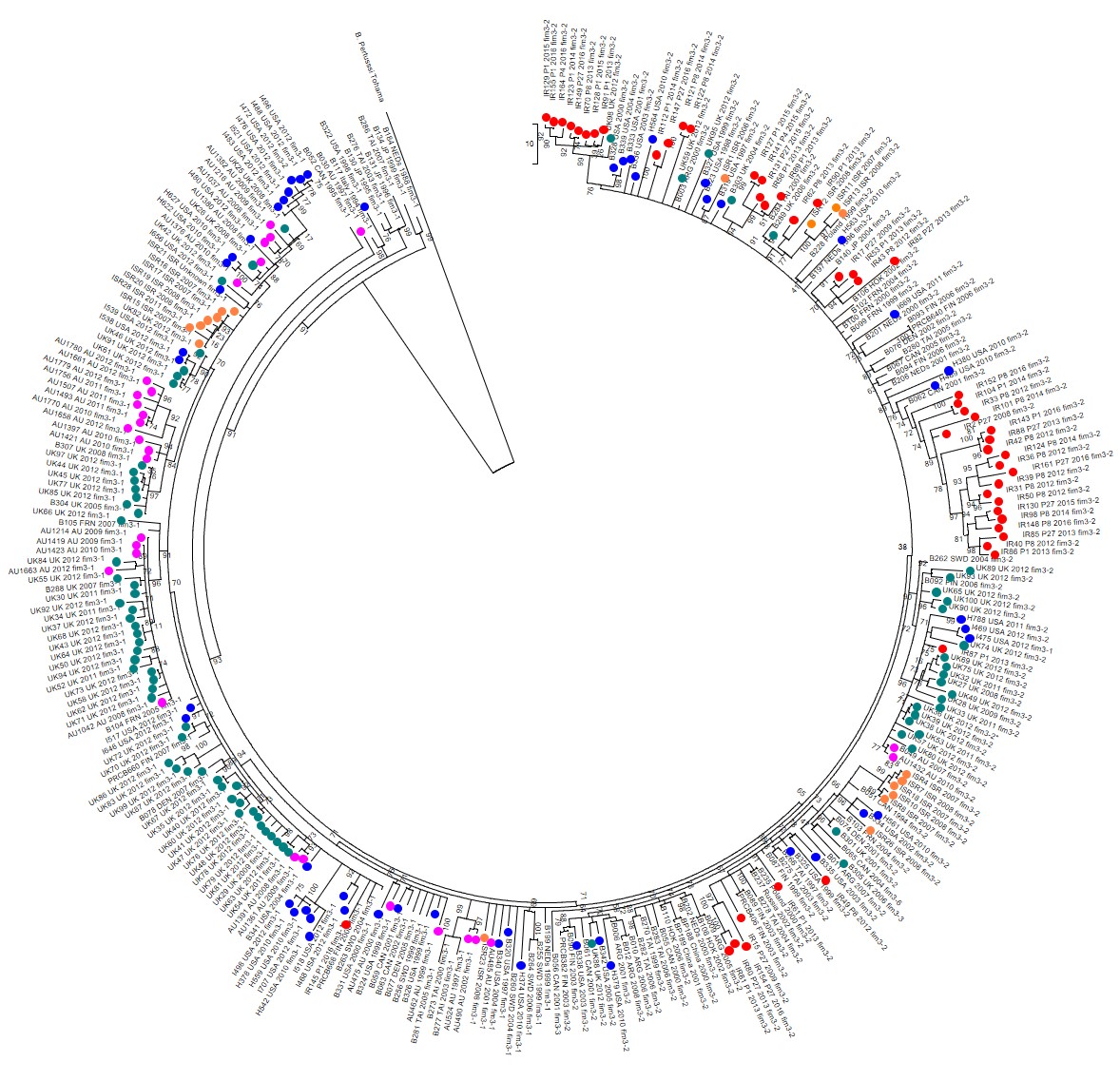

Supplement: Supplemental Material [file TEMI_A_1665479_SM4887.zip › Supplemetary-figure-1.jpg]
